# Supplementary material for: Superior ab initio identification, annotation and characterisation of TEs and segmental duplications from genome assemblies
Source: PLoS One. 2018 Mar 14;13(3):e0193588. doi: 10.1371/journal.pone.0193588 (PMC5851578; doi:10.1371/journal.pone.0193588)
Supplement: S1 Fig — Shows the coverage plot for the top 12 highest copy number (>2,000 copies) unclassified consensus sequences in the anole genome. (PDF) [file pone.0193588.s001.pdf]

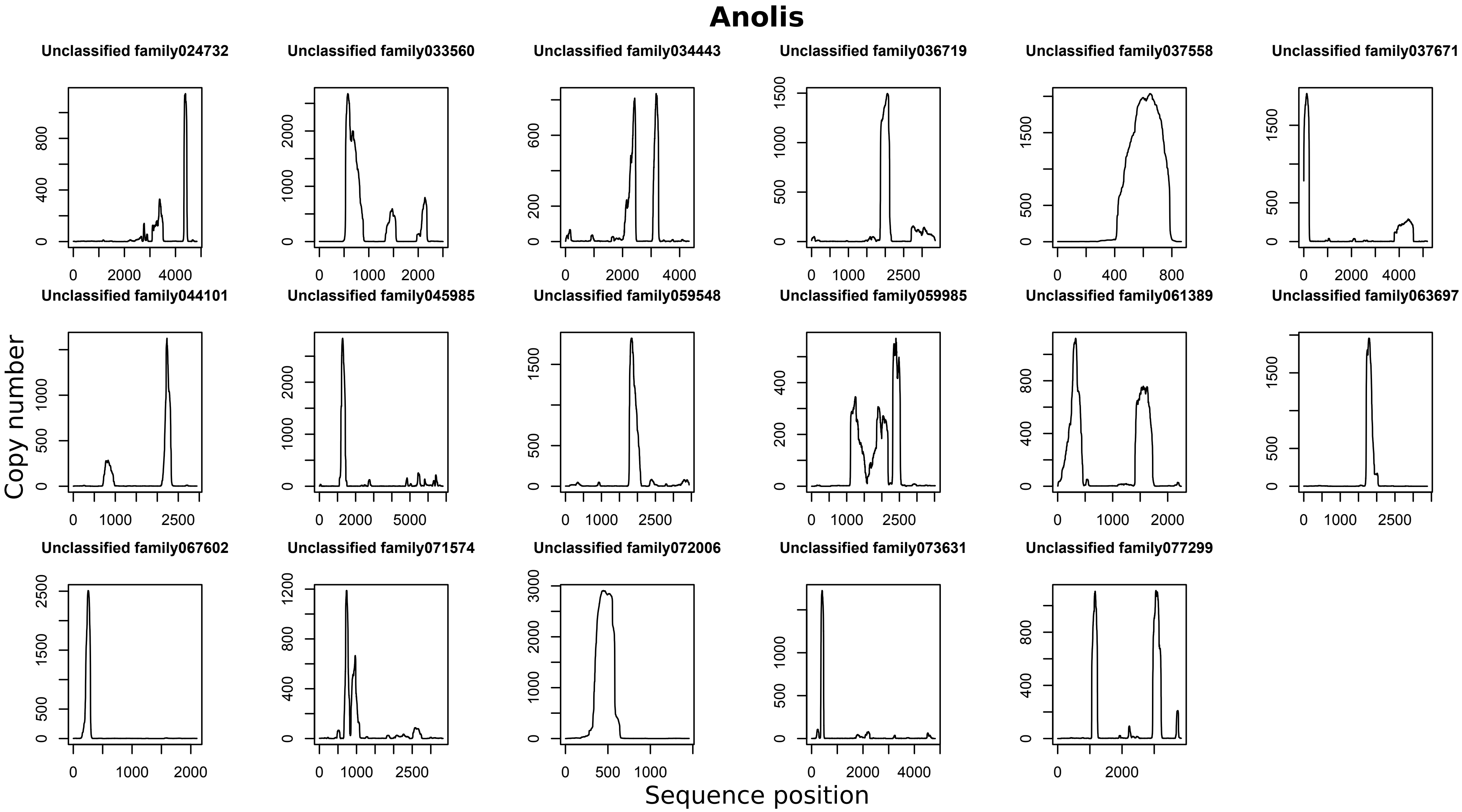

Anolis
Unclassified family024732
Unclassified family033560
Unclassified family034443
Unclassified family036719
Unclassified family037558
Unclassified family037671
Unclassified family044101
Unclassified family045985
Unclassified family059548
Unclassified family059985
Unclassified family061389
Unclassified family063697
Unclassified family067602
Unclassified family071574
Unclassified family072006
Unclassified family073631
Unclassified family077299
Copy number
Sequence position
