# Supplementary material for: Superior ab initio identification, annotation and characterisation of TEs and segmental duplications from genome assemblies
Source: PLoS One. 2018 Mar 14;13(3):e0193588. doi: 10.1371/journal.pone.0193588 (PMC5851578; doi:10.1371/journal.pone.0193588)
Supplement: S2 Fig — Shows the coverage plot for the top 21 highest copy number (>2,000 copies) unclassified consensus sequences in the opossum genome. (PDF) [file pone.0193588.s002.pdf]

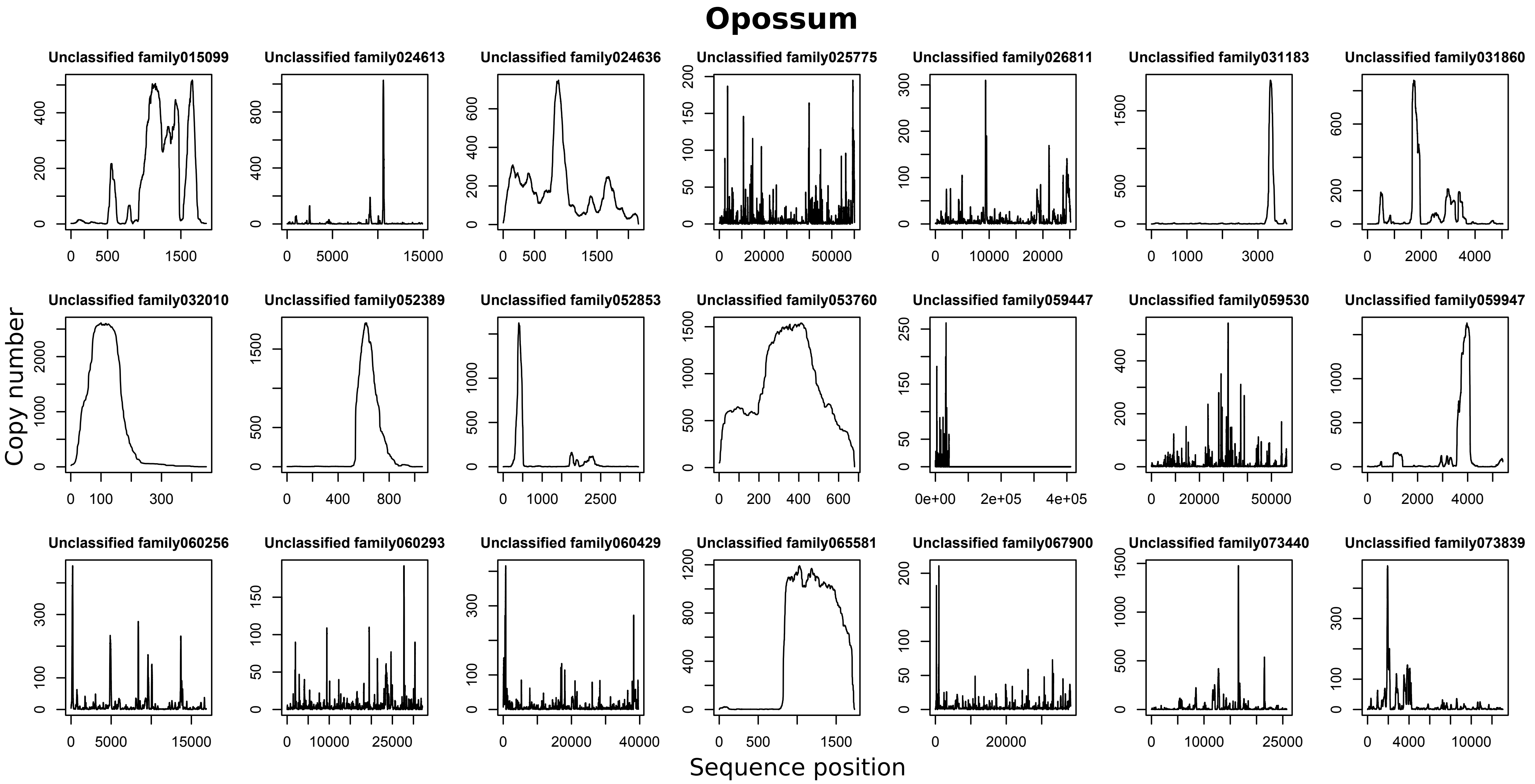
Opossum
Unclassified family015099
Unclassified family024613
Unclassified family024636
Unclassified family025775
Unclassified family026811
Unclassified family031183
Unclassified family031860
Unclassified family032010
Unclassified family052389
Unclassified family052853
Unclassified family053760
Unclassified family059447
Unclassified family059530
Unclassified family059947
Unclassified family060256
Unclassified family060293
Unclassified family060429
Unclassified family065581
Unclassified family067900
Unclassified family073440
Unclassified family073839
Copy number
Sequence position
