# Supplementary figures and images for: Superior ab initio identification, annotation and characterisation of TEs and segmental duplications from genome assemblies
Source: PLoS One. 2018 Mar 14;13(3):e0193588. doi: 10.1371/journal.pone.0193588 (PMC5851578; doi:10.1371/journal.pone.0193588)

# Human

## Coverage plot of unclassified family014307

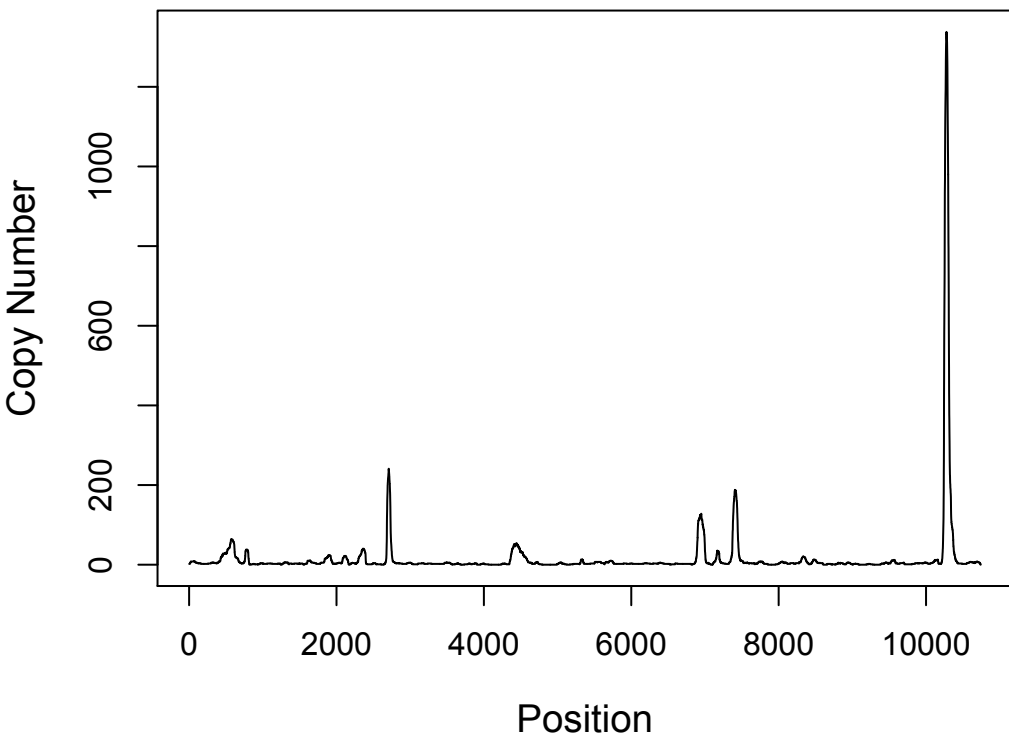

Supplement: S3 Fig — Shows the coverage plot for the highest copy number (>2,000 copies) unclassified consensus sequence in the human genome. (PDF) [file pone.0193588.s003.pdf]
