# Supplementary material for: Superior ab initio identification, annotation and characterisation of TEs and segmental duplications from genome assemblies
Source: PLoS One. 2018 Mar 14;13(3):e0193588. doi: 10.1371/journal.pone.0193588 (PMC5851578; doi:10.1371/journal.pone.0193588)
Supplement: S1 Appendix — Gives a detailed account of how to use our ab initio method to identify and annotate TEs from a genome assembly, including the benchmarks used for the seven species in this report. (PDF) [file pone.0193588.s004.pdf]

S1 Appendix

1 CARP manual 1.1

bíogo written by: Dan Kortschak
Document organised by: Lu Zeng
February 18, 2018

krishna and igor are *ab initio* repeat family identification and annotation programs, that identify repeat element boundaries and family relationships from whole-genome sequence data. These programs build, refine and classify consensus models of putative interspersed repeats. krishna and igor are built using bíogo (https://github.com/biogo/biogo/), a bioinformatics library for the Go language.
Disclaimer This document is provided to assist researchers with linux command line experience. We have done our best to provide usable instructions, examples and advice, but users assume full responsibility for the output they generate and the authors accept no responsibility for user generated output from any programs or methods listed herein.

For up to date versions of the carp documentation and code, please go to https://github.com/carp-te.

1.1 Prerequisites

1.1.1 Download Go

Available at (https://golang.org/dl).
For installation details, follow the instructions on the Go installation page.

1.1.2 Git

To perform the next step you will need Git to be installed. (Check that you have a git command before proceeding.)

If you do not have a working Git installation, follow the instructions on the Git download page.

1.1.3 Download bíogo Packages

Note: For convenience, add the workspace's bin subdirectory to your PATH, or add in $HOME/.profile.

```
export PATH=$PATH:$(go env GOPATH)/bin
```

Download and install krishna and igor packages from github.

```
go get -u github.com/biogo/examples/krishna
go get github.com/biogo/examples/krishna/matrix
go get github.com/biogo/examples/igor
go get github.com/biogo/examples/igor/seqer
go get github.com/biogo/examples/igor/gffer
```

1.1.4 Install CENSOR

Install censor to screen target genomes against a reference collection of repeats with masking symbols, as well as generating a report classifying all repeats found. CENSOR needs WU-BLAST/NCBI-BLAST and BioPerl installed.

CENSOR, along with instructions for installation, is available at CENSOR download page.

WU-BLAST can be downloaded at WU-BLAST download, NCBI-BLAST can be downloaded at ftp://ftp.ncbi.nlm.nih.gov/blast/executables/legacy.NOTSUPPORTED/2.2.26/. Note that we used NCBI-BLAST legacy code instead of the BLAST+ code because of the CENSOR dependency for that code. BioPerl can be downloaded at BioPerl download.

1.1.5 Install MUSCLE

Install MUSCLE to generate consensus sequences.
MUSCLE is available at MUSCLE download page. The installation information can be seen at MUSCLE Install page.

1.2 Example run

In this example, the human genome was downloaded as chromosomes (24 chromosomes) from UCSC into files called chr*.fa.

1.2.1 Use krishna to do pairwise alignment between human genome sequences

krishna-matrix helps you to align sequences by using a matrix table.

The default minimum hit length for krishna (-dplen) is 400bp, and minimum hit identity (-dpid) is 94%. The smaller the length and the lower the hit identity parameters you use, the more time and memory you will need.

If you want to change running parameters, for example, minimum hit length of 200bp, and minimum hit identity of 90%, just specify the parameters when running matrix -krishnaflags="-tmp=/scratch -threads=8 -log -filtid=0.9 -filtlen=200".

Now run the job (the krishna output files end with .gff):

```
cd /your/path/here/human
matrix -threads=8 -krishnaflags="-tmp=./ -threads=2 -log -filtid=0.94
 -filtlen=400" chr*.fa
```

-tmp: store the temporary files generated from running krishna, you can specify your own directory.
-threads (matrix): number of concurrent krishna instances to run. (default 6)
-threads (krishnaflags): number of threads to use for alignment. (default 1)
-filtid: minimum hit identity.

-filtlen: minimum hit length.

If genome sequence files are very big and consist of multiple contigs or scaffolds (>200MB), you can use bundle to split them into smaller files. For example,

```
go get github.com/biogo/examples/bundle
bundle -bundle 80000000 -in seq.fa
```

-bundle: specifies the total sequence length in a bundle. (default 20000000, 20MB).
-in: the genomes sequences you need to split.
Then run krishna job.

1.2.2 Use igor to report repeat feature family groupings in JSON format.

After running krishna, igor will take the pairwise alignment data to cluster repeat families.

```
find ./ -maxdepth 1 -name '[!.]*.gff' -print0 | xargs -r0 cat >
hg_krishna.gff
igor -in hg_krishna.gff -out hg94_krishna.json
```

1.2.3 Use seqer to generate consensus sequences from genome intervals .

seqer returns multiple fasta sequences corresponding to feature intervals described in the JSON output from igor.

gffer converts the JSON output of igor to gff. seqer will produce fastq consensus sequence output from either MUSCLE or MAFFT.

```
gffer < hg94_krishna.json > hg94_krishna.igor.gff
cat chr*.fa > hg19v37.mfa
seqer -aligner=muscle -dir=consensus -fasta=true -maxFam=100 -
subsample=true -minLen=0.95 -threads=12 -ref=hg19v37.mfa hg94_krishna.
igor.gff
```

-fasta: Output consensus as fasta with quality case filtering
-maxFam: maxFam indicates maximum family size permitted (0 == no limit).
-minLen: Minimum proportion of longest family member.
-threads: Number of concurrent aligner instances to run.

1.3 Benchmarks

Genome	Krishna Threads	Genome DB Size	Krishna run time (hh:mm)	Igor run time (hh:mm)	Seqer run time (hh:mm)
Human	8	3.0G	∼200	128:30	2:23
Bearded Dragon	8	1.8G	∼23	73:11	<4
Anolis	6	1.8G	76:52	97:32	2:40
Chicken	4	1017M	5	<4	<1
Opossum	8	3.5G	∼83	61:48	4:52
Platypus	8	2.0G	∼99	191:34	10:16

Analysis runs on a machine with 512GB RAM, running Red Hat Linux.

1.4 Repeat Library Annotation

Previous steps have generated repeat consensus sequences from the human genome, now we are going to annotate these repeat consensus sequences.
All the files used below can be found at
(https://data.mendeley.com/datasets/k88h5xnhcb/draft?a=d401233a-5af8-4879-81e8-c049b7133c8c).
All the code used below can be found at
(https://github.com/carp-te/carp-documentation/tree/master/code).

1.5 Annotate consensus sequences

Notes: For Java code used here you may need to specify the directories where your input data is and where you want your output written.

1.5.1 Annotate consensus sequences with repeat families.

Use censor to annotate consensus sequences with the Repbase library. The Vertebrates.fa we use here is the Repbase vertebrates repeat libraries downloaded on 1st March, 2016. You can download it from http://www.girinst.org/repbase/update/browse.php?type=All&format=FASTA&autonomous=on&nonautonomous=on&simple=on&division=Vertebrata&letter=A.

```
find ./consensus -maxdepth 1 -name '[!.]*.fq' -print0 | xargs -r0 cat >
 ConsensusSequences.fa

censor -bprm cpus=8 -lib ~/Vertebrates.fa -lib ~/our_known_reps_20130520.fasta
 ConsensusSequences.fa
```

For people that are not able to access WU-BLAST or prefer to use another aligner, CENSOR can also use NCBI BLAST instead (ftp://ftp.ncbi.nlm.nih.gov/blast/executables/blast+/LATEST/), please find details for CENSOR installation at CENSOR download page.

```
censor.ncbi -lib Vertebrates.fa -lib our_known_reps_20130520.fasta
 ConsensusSequences.fa
```

RepeatMasker can also be used to replace CENSOR in this step (RM-Blast was used as search engine).

```
cat Vertebrates.fa our_known_reps_20130520 > combined_library.fa

RepeatMasker -pa 16 -a -nolow -norna -dir ./ -lib combine_library.fa
 ConsensusSequences.fa

perl format_RMSK.pl ConsensusSequences.fa > tmp

mv tmp ConsensusSequences.fa
```

The censor output usually contains 5 files: ConsensusSequences.fa.map, ConsensusSequences.fa.aln, ConsensusSequences.fa.found, ConsensusSequences.fa.idx, ConsensusSequences.fa.masked.

1.5.2 Classify consensus sequences.

ConsensusSequences.fa and ConsensusSequences.fa.map are required in this step. You will also need to specify the directories for your input data and where you want your output written in the java code. Edit the source, compile and run.

```
javac ClassifyConsensusSequences.java
java ClassifyConsensusSequences
```

This should generate 5 output files: known.txt, partial.txt, check.txt, notKnown.fa, notknown.fa.gff. Then we need to further annotate these notKnown.fa consensus sequences.

1.5.3 Filter sequences.

This step contains three parts: 1) Identify potential protein sequences; 2) Identify GB_TE sequences; 3) Identify retrovirus sequences. You can run each part separately in parallel to save time. From these three steps, you will get three output files for following steps: 1) notKnown.fa.spwb.gff, 2) notKnown.fa.tewb.gff, 3) notKnown.fa.ervwb.gff.

First download uniprot protein dataset (uniprot_sprot.fasta.gz) from ftp://ftp.uniprot.org/pub/databases/uniprot/current_release/knowledgebase/complete/uniprot_sprot.fasta.gz.

Then download GB_TE dataset. First install EDirect from EDirect download page. Then download the data:

```
esearch -db protein -query "reverse transcriptase or transposon or repetitive element
    or RNA-directed DNA polymerase or pol protein or non-LTR retrotransposon or mobile
     element or retroelement or polyprotein or retrovirus or (group-specific antigen
    gag) or polymerase (pol)" | efetch -format fasta > 260118_GB_TE.fa
```

Retrovirus datasets can be downloaded from https://www.ncbi.nlm.nih.gov/genomes/GenomesGroup.cgi?taxid=11632.

Here are examples using WU-BLAST:

1) Identify potential protein sequences

```
gzip -d uniprot_sprot.fasta.gz

xdformat -p -k uniprot_sprot.fasta

blastx ./report_run/sprot notKnown.fa  -gspmax=1 -E 0.00001 -B 1 -V 1 -cpus=32 >
    notKnown.fa.spwb

python ./report_run/wublastx2gff.py notKnown.fa.spwb > notKnown.fa.spwb.gff
```

2) Identify GB_TE sequences

```
xdformat -p -k GB_TE.21032016.fa -o GB_TE.new

blastx ./BlastDB/GB_TE.new notKnown.fa -gspmax=1 -E 0.00001 -B 1 -V 1 -cpus=32 >
    notKnown.fa.tewb

python ./report_run/wublastx2gff.py notKnown.fa.tewb > notKnown.fa.tewb.gff
```

3) Identify potential retrovirus sequences

```
xdformat -n -k all_retrovirus.fasta

tblastx ./BlastDB/all_retrovirus.fasta notKnown.fa -gspmax=1 -E 0.00001 -B 1 -V 1 \
-cpus=32 > notKnown.fa.ervwb

python ./report_run/wublastx2gff.py notKnown.fa.ervwb > notKnown.fa.ervwb.gff
```

-gspmax: max. number of gapped HSPs (GSPs) saved per subject sequence (default 0; 0 => unlimited).
-B -V: the B and V options limit the number of subject sequences for which any results whatsoever are reported, regardless of the number of HSPs or GSPs found.
-E: Expectation value (E) threshold for saving hits.
-cpus: no. of processors to utilize on multi-processor systems.

If you wish to use NCBI-BLAST, we tested different NCBI-BLAST parameters, to make the results as consistent as possible to the WU-BLAST results. See below for examples using NCBI-BLAST:

1) Identify potential protein sequences

```
makeblastdb -in uniprot_sprot.fasta -dbtype prot

blastx -db uniprot_sprot.fasta -query notKnown.fa -max_hsps 1 -seg no -evalue 0.00001 -num_threads 32 -max_target_seqs 1 -word_size 2 -outfmt 6 -out notKnown.fa.spwb.ncbi

awk '{print $1"\t""blast""\t""hit""\t"$7"\t"$8"\t"$11"\t"".""\t"".""\t""Target sp|"
$2" "$9" "$10}' notKnown.fa.spwb.ncbi > tmp

awk '{if($4>$5) print $1"\t"$2"\t"$3"\t"$5"\t"$4"\t"$6"\t"$7"\t"$8"\t"$9" "$10" "$11
" "$12; else print $0}' tmp > notKnown.fa.spwb.gff
```

2) Identify GB_TE sequences

```
makeblastdb -in GB_TE.21032016.fa -dbtype prot -out GB_TE.new

blastx -db GB_TE.new -query notKnown.fa -max_hsps 1 -seg no -evalue 0.00001 \
-num_threads 32 -max_target_seqs 1 -word_size 2 -outfmt 6 -out notKnown.fa.tewb.ncbi

awk '{print $1"\t""blast""\t""hit""\t"$7"\t"$8"\t"$11"\t"".""\t"".""\t""Target sp|"
$2" "$9" "$10}' notKnown.fa.ervwb.ncbi > tmp

awk '{if($4>$5) print $1"\t"$2"\t"$3"\t"$5"\t"$4"\t"$6"\t"$7"\t"$8"\t"$9" "$10" "$11
" "$12; else print $0}' tmp > notKnown.fa.tewb.gff
```

3) Identify potential retrovirus sequences

```
makeblastdb -in all_retrovirus.fasta -dbtype nucl

tblastx -db all_retrovirus.fasta -query notKnown.fa -max_hsps 1 -seg no -evalue 0.00001 -num_threads 32 -max_target_seqs 1 -word_size 2 -outfmt 6 -out notKnown.fa.ervwb.ncbi

awk '{print $1"\t""blast""\t""hit""\t"$7"\t"$8"\t"$11"\t"".""\t"".""\t""Target sp|"
$2" "$9" "$10}' notKnown.fa.ervwb.ncbi > tmp

awk '{if($4>$5) print $1"\t"$2"\t"$3"\t"$5"\t"$4"\t"$6"\t"$7"\t"$8"\t"$9" "$10" "$11
" "$12; else print $0}' tmp > notKnown.fa.ervwb.gff
```

-db: BLAST database name
-query: Input file name
-max_hsps: Set maximum number of HSPs per subject sequence to save for each query, NCBI-BLAST doesn't have gsps option, but we tested with hsps in WU-BLAST, the result remain almost the same
-seg: Filter query sequence with SEG (Format: 'yes', 'window locut hicut', or 'no' to disable), default WU-BLAST is off
-evalue: Expectation value (E) threshold for saving hits
-num_threads: Number of threads (CPUs) to use in the BLAST search
-max_target_seqs: Maximum number of aligned sequences to keep
-word_size: Word size for wordfinder algorithm.

1.5.4 Get protein information from consensus sequences.

Another java program GetProteins.java will be used. It needs two input files: notKnown.fa, notKnown.fa.spwb.gff (Generated from previous step).

```
javac GetProteins.java
java GetProteins
```

You will get 2 output files: proteins.txt (a list of families that have been identified as proteins and the proteins they match); notKnownNotProtein.fa (a fasta file of the families that were not classified).

1.5.5 Check for simple sequence repeats (SSR).

Check for existence of SSR in the unknown sequences, using phobos. Phobos can be downloaded at (http://www.ruhr-uni-bochum.de/ecoevo/cm/cm_phobos.htm). We used executable: phobos-linux-gcc4.1.2.

```
phobos-linux-gcc4.1.2 -r 7 --outputFormat 0 --printRepeatSeqMode 0
 notKnownNotProtein.fa > notKnownNotProtein.phobos
```

1.5.6 Identify the sequences that are SSRs from the phobos output.

phobos output will be used to identify SSRs: notKnownNotProtein.phobos.

```
javac IdentifySSRs.java
java IdentifySSRs
```

Your output will be a file called: SSR.txt

1.5.7 Generate annotated repeat library.

There are ten input files that are required to generate a repeat library:
1. ConsensusSequences.fa
2. ConsensusSequences.fa.map
3. notKnown.fa.tewb.gff
4. notKnown.fa.ervwb.gff
5. protein.txt
6. known.txt
7. GB_TE.21032016.fa
8. all_retrovirus.fasta
9. SSR.txt (if you do not have this, leave the definition in, it will generate error messages, but will not stop the program or affect the results.)
10. LA4v2-satellite.fa (you do not have this, or equivalent, as you didn't have any satellites, but leave the definition in-it will cause error messages, but will not stop the program or affect the results.)

```
javac GenerateAnnotatedLibrary.java
java GenerateAnnotatedLibrary
```

This will generate a library called "Human_Repeat_Library.fasta", you can rename this file to whatever you want.

1.6 Benchmarks2

Genome	Consensus sequences size	Censor first run time (hh:mm)	reportJ.sh (hh:mm)	phobos run time (hh:mm)
Human	38M	5:14	19:30	<00:10
Bearded Dragon	88M	22:21	<178	<00:10
* New Bearded Dragon	88M	7:13	18:28	<00:10
Anolis	63M	9:42	78	<00:10
Chicken	18M	3:01	<24	<00:10
Opossum	60M	13:17	80	<01:00
Platypus	162M	17:34	115	<01:00

Analysis run on a slurm machine with 4∼16 cpus, and 8GB RAM, running Red Hat Linux.
* New Bearded dragon analysis used same bearded dragon genome, except it was run on a High Performance Computing machine with 32 cpus, running Red Hat Linux.
