# Supplementary material for: Superior ab initio identification, annotation and characterisation of TEs and segmental duplications from genome assemblies
Source: PLoS One. 2018 Mar 14;13(3):e0193588. doi: 10.1371/journal.pone.0193588 (PMC5851578; doi:10.1371/journal.pone.0193588)
Supplement: S1 Table — Shows the systematic name, common name, genome version, source and submitter for all the genomes tested for our ab initio method. (PDF) [file pone.0193588.s005.pdf]

No	Systematic Name	Common Name	Genome Version	Source	Submitter
1	*Homo sapiens*	Human	GRCh37(hg19)	NCBI	GRC
2	*Central Pogona Vitticeps*	Bearded Dragon	Pogona_vitticeps.male	NCBI	BRAEMBL
3	*Anolis Carollinensis*	Anolis lizard	PanoCar2	NCBI	Broad
4	*Gallus gallus*	Chicken	galGal4	NCBI	ICGS
5	*Monodelphis domestica*	Opossum	monDom5	NCBI	GAT
6	*Ornithorhynchus anatinus*	Platypus	ornAna1	NCBI	WashU

The Following abbreviations are used for submitters:
Genome Sequencing Platform, The Genome Assembly Team = GAT;
Genome Reference Consortium = GRC;
International Chicken Genome Consortium = ICGS;
Washington University = WashU.
