# Supplementary material for: Superior ab initio identification, annotation and characterisation of TEs and segmental duplications from genome assemblies
Source: PLoS One. 2018 Mar 14;13(3):e0193588. doi: 10.1371/journal.pone.0193588 (PMC5851578; doi:10.1371/journal.pone.0193588)
Supplement: S2 Table — Shows the systematic name, total sequence length, scaffold N50, contig N50 and assembly level. (PDF) [file pone.0193588.s006.pdf]

No	Species	Total Sequence Length	Scaffold N50	Contig N50	Assemble Level
1	*Homo sapiens*	3,095,677,412	44,983,201	38,440,852	Chromosome
2	*Pogona Vitticeps*	1,716,675,060	2,477,614	-	Scaffold
3	*Anolis Carollinensis*	1,799,143,587	4,033,265	79,867	Chromosome
4	*Gallus gallus*	1,046,932,099	12,877,381	279,750	Chromosome
5	*Monodelphis domestica*	3,605,631,728	59,809,810	108,014	Chromosome
6	*Ornithorhynchus anatinus*	2,073,148,626	958,970	11,554	Chromosome
