# Supplementary material for: Superior ab initio identification, annotation and characterisation of TEs and segmental duplications from genome assemblies
Source: PLoS One. 2018 Mar 14;13(3):e0193588. doi: 10.1371/journal.pone.0193588 (PMC5851578; doi:10.1371/journal.pone.0193588)
Supplement: S3 Table — Shows the systematic name, assembly method, sequencing techonology and estimated genome coverage for the seven genomes in this study. (PDF) [file pone.0193588.s007.pdf]

No	Species	Assembly Method	Sequencing Technology	Genome Coverage
1	*Homo sapiens*	Celera	Sanger	20x
2	*Pogona Vitticeps*	SOAP deNovo	Illumina HiSeq 2000	85.5x
3	*Anolis Carollinensis*	Arachne v.3.0.0	ABI	7.10x
4	*Gallus gallus*	Celera Assemblerv.5.4	Sanger; 454	12x
5	*Monodelphis domestica*	ARACHNE2+	Sanger	6.8x
6	*Ornithorhynchus anatinus*	PCAP	WGS plasmid, fosmid end and BAC end sequences	6x
