# Supplementary material for: Superior ab initio identification, annotation and characterisation of TEs and segmental duplications from genome assemblies
Source: PLoS One. 2018 Mar 14;13(3):e0193588. doi: 10.1371/journal.pone.0193588 (PMC5851578; doi:10.1371/journal.pone.0193588)
Supplement: S4 Table — Here we show the compute time used for the seven tested species with CARP and RMD. (PDF) [file pone.0193588.s008.pdf]

	**Chicken**		**Bearded dragon**		**Anolis**		**Platypus**		**Opossum**		**Human**	
	De novo	RMD	*De novo*	RMD	*De novo*	RMD	*De novo*	RMD	*De novo*	RMD	*De novo*	RMD
Time consumed (h)	37	8	276	39	266	19	434	49	244	21	495	18
