# Supplementary material for: Superior ab initio identification, annotation and characterisation of TEs and segmental duplications from genome assemblies
Source: PLoS One. 2018 Mar 14;13(3):e0193588. doi: 10.1371/journal.pone.0193588 (PMC5851578; doi:10.1371/journal.pone.0193588)
Supplement: S5 Table — Total length (bp) of consensus sequence libraries generated by CARP and RMD. (PDF) [file pone.0193588.s009.pdf]

	Chicken		Bearded dragon		Anolis		Platypus		Opossum		Human	
	CARP	RMD	CARP	RMD	CARP	RMD	CARP	RMD	CARP	RMD	CARP	RMD
Well -annotated	4,100,530	154,743	6,758,949	396,987	21,498,947	564,031	4,022,323	204,475	28,379,922	651,545	10,914,900	462,921
Unclassified	10,871,250	20,553	42,128,582	220,612	34,790,047	166,735	142,361,947	40,446	25,672,894	66,497	20,779,056	2,018
Total	14,971,780	175,296	48,887,531	617,599	56,288,994	730,766	146,384,270	244,921	54,052,816	718,042	31,693,956	464,939
