# Supplementary material for: Superior ab initio identification, annotation and characterisation of TEs and segmental duplications from genome assemblies
Source: PLoS One. 2018 Mar 14;13(3):e0193588. doi: 10.1371/journal.pone.0193588 (PMC5851578; doi:10.1371/journal.pone.0193588)
Supplement: S6 Table — Comparison of the total consensus sequence lengths (bp) of specific TE types generated by CARP and RMD. (PDF) [file pone.0193588.s010.pdf]

	Chicken		Bearded dragon		Anolis		Platypus		Opossum		Human	
	CARP	RMD	CARP	RMD	CARP	RMD	CARP	RMD	CARP	RMD	CARP	RMD
SINE	0	542	438,341	13,932	705,660	14,210	620,446	42,051	217,725	43,274	3,516,601	21,660
LINE	2,504,529	71,746	5,710,722	217,145	13,287,702	288,130	2,803,327	101,511	17,085,608	364,368	5,115,349	249,096
LTR	1,364,464	56,279	125,606	78,822	4,034,353	121,054	170,087	24,980	10,832,916	212,088	2,028,463	134,103
DNA	31,724	9,635	480,443	79,404	3,461,618	120,828	20,525	14,770	230,518	25,880	76,177	42,308
Others	199,813	16,541	3,837	7,684	9,614	19,809	407,938	21,163	13,155	5,935	178,310	15,754
Total	4,100,530	154,743	6,758,949	396,987	21,498,947	564,031	4,022,323	204,475	28,379,922	651,545	10,914,900	462,921
