# Supplementary material for: Superior ab initio identification, annotation and characterisation of TEs and segmental duplications from genome assemblies
Source: PLoS One. 2018 Mar 14;13(3):e0193588. doi: 10.1371/journal.pone.0193588 (PMC5851578; doi:10.1371/journal.pone.0193588)
Supplement: S7 Table — Shows the copy number, total base pairs (bp) and the percentage of specific repeat class in the chicken genome. (PDF) [file pone.0193588.s011.pdf]

Group	Copy number	Total bp	Percentage coverage of genome
**Non-LTR retrotransposons**			
**LINEs**			
CR1	263,953	75,352,619	7.197
Tx1	19,284	1,282,829	0.123
LINE L2	10,268	991,208	0.094
SINE	9,766	957,771	0.092
Others	46,883	3,327,936	0.318
	350,154	81,912,363	7.824
**DNA transposons**			
hAT	64,915	5,762,565	0.550
Mariner	35,785	5,210,866	0.498
Charlia	9,267	3,941,040	0.376
DNA	37,393	3,612,850	0.345
Others	120,170	8,667,690	0.828
	267,530	27,195,011	2.597
**LTR**			
Copia	139,422	9,641,970	0.921
Gypsy	32,502	2,180,147	0.208
BEL	23,661	1,450,356	0.138
Others	26,536	3,756,055	0.359
	222,121	17,028,528	1.626
**ERVs**			
GGLTR	18,844	7,093,568	0.677
Others	72,068	11,146,021	1.065
	90,912	18,239,589	1.742
SSR	38,969	4,172,125	0.399
Others	112,310	11,734,743	1.121
**Well-annotated**	1,081,996	160,282,359	15.309
**Unknown**	84,926	20,806,411	1.987
**Total**	1,166,922	181,088,770	17.296
