# Supplementary material for: Superior ab initio identification, annotation and characterisation of TEs and segmental duplications from genome assemblies
Source: PLoS One. 2018 Mar 14;13(3):e0193588. doi: 10.1371/journal.pone.0193588 (PMC5851578; doi:10.1371/journal.pone.0193588)
Supplement: S8 Table — Shows the copy number, total base pairs (bp) and the percentage of specific repeat class in the anolis genome. (PDF) [file pone.0193588.s012.pdf]

Group	Copy number	Total bp	Percentage coverage of genome
**Non-LTR retrotransposons**			
**LINEs**			
CR1	206,414	58,082,752	3.228
LINE L2	282,443	75,470,209	4.195
LINE L1	159,992	35,267,057	1.960
RTE	97,078	32,188,630	1.789
R4	65,406	24,750,832	1.376
Others	184,317	37,754,227	2.098
	995,650	263,513,707	14.646
**SINEs**			
SINE-2	344,568	71,189,836	3.957
Others	9,136	1,110,966	0.062
	353,704	72,300,802	4.019
**DNA transposons**			
hAT	526,130	77,476,809	4.307
Mariner	409,901	67,917,600	3.775
Helitron	179,050	35,682,369	1.983
DNA	300,556	39,263,225	2.182
Others	112,203	10,576,832	0.588
	1,527,840	230,916,835	12.835
**LTR**			
Gypsy	241,711	67,543,777	3.754
Copia	53,434	8,461,466	0.470
DIRS	24,293	9,446,400	0.525
Others	43,490	9,248,301	0.514
	362,928	94,699,944	5.263
**ERVs**			
ERV1/2/3	79,860	12,977,630	0.720
**SSR**	72,160	8,040,704	0.447
**Others**	250,255	19,205,549	1.067
**Well-annotated**	3,642,397	701,655,171	38.997
**Unknown**	1,678,936	216,304,228	12.022
**Total**	5,321,333	917,959,399	51.019
