# Supplementary material for: Superior ab initio identification, annotation and characterisation of TEs and segmental duplications from genome assemblies
Source: PLoS One. 2018 Mar 14;13(3):e0193588. doi: 10.1371/journal.pone.0193588 (PMC5851578; doi:10.1371/journal.pone.0193588)
Supplement: S9 Table — Shows the copy number, total base pairs (bp) and the percentage of specific repeat class in the bearded dragon genome. (PDF) [file pone.0193588.s013.pdf]

Group	Copy number	Total bp	Percentage coverage of genome
**Non-LTR retrotransposons**			
**LINEs**			
LINE-2	229,080	40,358,815	2.351
RTE(BovB)	222,363	57,590,102	3.355
RTE	124,644	26,011,403	1.515
LINE(CR1)	194,333	35,912,124	2.092
LINE-1	126,975	15,181,332	0.884
Penelope	95,133	11,527,155	0.671
Other	96,331	12,761,752	0.744
	1,088,859	199,342,683	11.612
**SINEs**			
SINE-2	206,966	28,277,402	1.647
Other	17,391	1,252,811	0.073
	224,357	29,530,213	1.720
**DNA transposons**			
Mariner	201,988	22,220,773	1.294
hAT	260,256	23,832,751	1.388
others	215,581	14,924,598	0.870
	677,825	60,978,122	3.552
**LTR**			
DIRS	62,273	11,501,289	0.670
Gypsy	207,875	15,163,491	0.883
Copia	74,482	5,108,316	0.298
other	45,829	2,782,279	0.162
	390,459	34,555,375	2.013
**ERVs**			
ERV1/2/3	99,037	6,371,060	0.371
SSR	83,878	8,278,572	0.482
Others	217,184	15,519,668	0.905
**Well-annotated**	2,781,599	354,575,693	20.655
**Unknown**	2,882,556	381,401,912	22.217
**Total**	5,664,155	735,977,605	42.872
