# Supplementary material for: Superior ab initio identification, annotation and characterisation of TEs and segmental duplications from genome assemblies
Source: PLoS One. 2018 Mar 14;13(3):e0193588. doi: 10.1371/journal.pone.0193588 (PMC5851578; doi:10.1371/journal.pone.0193588)
Supplement: S10 Table — Shows the copy number, total base pairs (bp) and the percentage of specific repeat class in the platypus genome. (PDF) [file pone.0193588.s014.pdf]

Group	Copy number	Total bp	Percentage coverage of genome
**Non-LTR retrotransposons**			
**LINEs**			
LINE L2	2,141,746	380,315,682	18.345
CR1	105,713	16,510,780	0.796
BovB	14,412	3,793,080	0.183
Others	156,741	22,358,028	1.078
	2,418,612	422,977,570	20.402
**SINEs**			
Mon1	2,039,591	376,397,912	18.156
PlatSINE	49,237	11,920,732	0.575
Others	152,447	16,041,435	0.774
	2,241,275	404,360,079	19.505
**DNA transposons**			
hAT	105,794	10,839,474	0.523
Mariner	90,510	12,440,134	0.600
Others	163,790	12,469,529	0.601
	360,094	35,749,137	1.724
**LTR**			
Copia	35,510	2,515,002	0.121
Gypsy	150,673	10,227,632	0.493
Other	58,994	6,189,423	0.299
	245,177	18,932,057	0.913
**ERVs**			
ERV	85,721	8,813,118	0.425
SSR	107,515	13,599,686	0.656
Others	364,116	46,404,979	2.238
**Well-annotated**	5,822,510	950,836,626	45.863
**Unknown**	2,321,717	261,289,414	12.603
**Total**	8,144,227	1,212,126,040	58.466
