# Supplementary material for: Superior ab initio identification, annotation and characterisation of TEs and segmental duplications from genome assemblies
Source: PLoS One. 2018 Mar 14;13(3):e0193588. doi: 10.1371/journal.pone.0193588 (PMC5851578; doi:10.1371/journal.pone.0193588)
Supplement: S11 Table — Shows the copy number, total base pairs (bp) and the percentage of specific repeat class in the opossum genome. (PDF) [file pone.0193588.s015.pdf]

Group	Copy number	Total bp	Percentage coverage of genome
**Non-LTR retrotransposons**			
**LINEs**			
LINE L1	1,252,041	703,738,862	19.518
CR1	1,096,864	225,839,324	6.264
RTE	220,608	73,141,920	2.028
LINE2	50865	5042973	0.140
Others	127,564	9,278,127	0.257
	2,747,942	1,017,041,206	28.206
**SINEs**			
SINE-1	506,241	88,145,053	2.444
SINE MIR	547,244	67,502,440	1.872
THER	597,652	89,262,998	2.476
Others	746,517	131,606,069	3.650
	2,397,654	376,516,560	10.442
**DNA transposons**			
hAT	350,343	44,822,893	1.243
Mariner	157,450	23,453,500	0.651
Charlia	17,487	4,683,795	0.130
Others	439,658	37,393,312	1.037
	964,938	110,353,500	3.061
**LTR**			
Copia	60,653	4,045,526	0.112
Gypsy	289,603	20,968,390	0.582
Others	100,662	9,982,015	0.277
	450,918	34,995,931	0.971
**ERVs**			
ERV	772,823	347,807,695	9.646
SSR	245,921	34,883,702	0.967
Others	302,420	27,218,434	0.755
**Well-annotated**	7,882,616	1,948,817,028	54.049
**Unknown**	1,164,500	141,831,766	3.934
**Total**	9,047,116	2,090,648,794	57.983
