# Supplementary material for: Superior ab initio identification, annotation and characterisation of TEs and segmental duplications from genome assemblies
Source: PLoS One. 2018 Mar 14;13(3):e0193588. doi: 10.1371/journal.pone.0193588 (PMC5851578; doi:10.1371/journal.pone.0193588)
Supplement: S12 Table — Shows the copy number, total base pairs (bp) and the percentage of specific repeat class in the human genome. (PDF) [file pone.0193588.s016.pdf]

Group	Copy number	Total bp	Percentage coverage of genome
**Non-LTR retrotransposons**			
**LINEs**			
LINE L1	1,061,429	504,783,605	16.306
CR1	348,909	65,998,881	2.132
LINE L2	31,074	2,741,726	0.089
Others	135,468	11,057,731	0.357
	1,576,880	584,581,943	18.884
**SINEs**			
Alu	1,148,493	285,614,276	9.226
MIR	367,336	49,924,630	1.613
Others	91,907	12,580,275	0.406
	1,607,736	348,119,181	11.245
**DNA transposons**			
hAT	408,174	60,848,627	1.966
Mariner	191,198	42,157,411	1.362
Others	386,094	36,070,514	1.165
	985,466	13,907,6552	4.493
**LTR**			
Gypsy	279,091	20,266,383	0.655
THE1	10,979	11,686,418	0.377
Copia	55,336	3,810,198	0.123
Others	90,172	11,450,442	0.370
	435,578	47,213,441	1.525
**ERVs**			
ERV	802,941	246,168,345	7.952
SSR	147,561	18,249,839	0.590
Others	294,098	34,097,043	1.101
**Well-annotated**	5,850,260	1,417,506,344	45.790
**Unknown**	762,391	91,054,381	2.941
**Total**	6,612,651	1,508,560,725	48.731
