# Supplementary material for: Superior ab initio identification, annotation and characterisation of TEs and segmental duplications from genome assemblies
Source: PLoS One. 2018 Mar 14;13(3):e0193588. doi: 10.1371/journal.pone.0193588 (PMC5851578; doi:10.1371/journal.pone.0193588)
Supplement: S13 Table — Shows the estimate value, standard error, t-value, p-value and significance codes from linear regression analysis. Significance asterisks follow the conventions of R, i.e. ***, **, *, ., for p-values below 0.001, 0.01, 0.05 and 0.1 respectively. (PDF) [file pone.0193588.s017.pdf]

			Estimate	Std. Error	T	Pr(>|t|)	Significance
Chicken	*de novo*	Intercept	-1.055	0.037	-28.49	<2e-16	***
		Length	0.689	0.013	53.44	<2e-16	***
	RMD	Intercept	-1.690	0.424	-3.911	0.000148	***
		Length	1.226	0.160	7.653	4.18e-12	***
Bearded Dragon	*de novo*	Intercept	-1.690	0.016	-105.2	<2e-16	***
		Length	1.035	0.006	164.5	<2e-16	***
	RMD	Intercept	1.264	0.123	10.27	<2e-16	***
		Length	0.713	0.053	13.54	<2e-16	***
Anolis	*de novo*	Intercept	-1.822	0.030	-60.40	<2e-16	***
		Length	1.005	0.011	92.78	<2e-16	***
	RMD	Intercept	1.147	0.178	6.435	1.75e-10	***
		Length	0.501	0.074	6.753	2.20e-11	***
Platypus	*de novo*	Intercept	-2.156	0.009	-245.0	<2e-16	***
		Length	1.098	0.003	360.7	<2e-16	***
	RMD	Intercept	-0.329	0.218	-1.51	0.132	
		Length	1.026	0.092	11.10	<2e-16	***
Opossum	*de novo*	Intercept	-0.867	0.032	-26.75	<2e-16	***
		Length	0.700	0.011	61.49	<2e-16	***
	RMD	Intercept	-0.709	0.286	-2.477	0.0135	*
		Length	0.974	0.114	8.526	<2e-16	***
Human	*de novo*	Intercept	-1.733	0.021	-80.7	<2e-16	***
		Length	1.047	0.008	135.1	<2e-16	***
	RMD	Intercept	-0.290	0.248	-1.168	0.244	
		Length	0.617	0.101	6.122	2.05e-09	***
